# IL-7-Induced Proliferation of Human Naive CD4 T-Cells Relies on Continued Thymic Activity

**DOI:** 10.3389/fimmu.2017.00020

**Published:** 2017-01-19

**Authors:** Susana L. Silva, Adriana S. Albuquerque, Paula Matoso, Bénédicte Charmeteau-de-Muylder, Rémi Cheynier, Dário Ligeiro, Miguel Abecasis, Rui Anjos, João T. Barata, Rui M. M. Victorino, Ana E. Sousa

**Affiliations:** ^1^Faculdade de Medicina, Instituto de Medicina Molecular, Universidade de Lisboa, Lisboa, Portugal; ^2^Centro Hospitalar de Lisboa Norte, Hospital de Santa Maria, Lisboa, Portugal; ^3^Cytokines and Viral Infections, Immunology Infection and Inflammation Department, Institut Cochin, INSERM, U1016, Paris, France; ^4^CNRS, UMR8104, Paris, France; ^5^Université Paris Descartes, Paris, France; ^6^Centro de Sangue e Tranplantação de Lisboa, Instituto Português de Sangue e Transplantação, IP, Lisboa, Portugal; ^7^Departamento do Coração, Hospital de Santa Cruz, Centro Hospitalar de Lisboa Ocidental, Carnaxide, Portugal

**Keywords:** naive CD4 T-cells, T-cell homeostasis, IL-7, thymus, thymectomy

## Abstract

Naive CD4 T-cell maintenance is critical for immune competence. We investigated here the fine-tuning of homeostatic mechanisms of the naive compartment to counteract the loss of *de novo* CD4 T-cell generation. Adults thymectomized in early childhood during corrective cardiac surgery were grouped based on presence or absence of thymopoiesis and compared with age-matched controls. We found that the preservation of the CD31^−^ subset was independent of the thymus and that its size is tightly controlled by peripheral mechanisms, including prolonged cell survival as attested by Bcl-2 levels. Conversely, a significant contraction of the CD31^+^ naive subset was observed in the absence of thymic activity. This was associated with impaired responses of purified naive CD4 T-cells to IL-7, namely, *in vitro* proliferation and upregulation of CD31 expression, which likely potentiated the decline in recent thymic emigrants. Additionally, we found no apparent constraint in the differentiation of naive cells into the memory compartment in individuals completely lacking thymic activity despite upregulation of *DUSP6*, a phosphatase associated with increased TCR threshold. Of note, thymectomized individuals featuring some degree of thymopoiesis were able to preserve the size and diversity of the naive CD4 compartment, further arguing against complete thymectomy in infancy. Overall, our data suggest that robust peripheral mechanisms ensure the homeostasis of CD31^−^ naive CD4 pool and point to the requirement of continuous thymic activity to the maintenance of IL-7-driven homeostatic proliferation of CD31^+^ naive CD4 T-cells, which is essential to secure T-cell diversity throughout life.

## Introduction

Long-term preservation of the naive CD4 T-cell pool is vital to ensure immunity to foreign antigens and to maintain peripheral tolerance (1, 2). Naive CD4 T-cells are preserved throughout life due to a dynamic balance between thymic generation, and peripheral proliferation, survival, death, or differentiation into memory/effector cells (1, 3).

The thymus is known to be functional up to the sixth decade of life, even though an age-dependent decline in thymic activity occurs (4). Thymic output can be estimated through the quantification of TCR rearrangement circles (TRECs), which are excision by-products generated during T-cell development in the thymus (4). These episomal DNA fragments are progressively diluted with cell division in the periphery (5), being thus enriched in recent thymic emigrant cells (RTEs). Aging is associated with a progressive reduction in TREC levels (4). This decline is much more striking than the one observed in naive CD4 T-cell counts, indicating that the loss of cell replenishment due to thymic involution is complemented by peripheral dilution due to cell proliferation (4, 6–8). In fact, in contrast to mice, the establishment and maintenance of the human naive CD4 T-cell compartment are currently thought to significantly rely on post-thymic T-cell proliferation (3, 9, 10). Several *in silico* studies suggest that thymic output *per se* is insufficient to guarantee the size of the peripheral naive T-cell compartment without a major contribution of cell proliferation in the periphery (3, 9, 10). This homeostatic proliferation is driven by self-peptide/MHC interaction and/or cytokines, namely, IL-7 (9, 11, 12).

IL-7 is essential for thymopoiesis and plays a key role in peripheral naive T-cell survival through the induction of Bcl-2 (11, 13). In addition, IL-7 induces low-level naive T-cell proliferation (11, 13), which is particularly important in lymphopenic clinical settings (11, 13). In steady-state conditions, this homeostatic proliferation within the naive CD4 T-cell compartment is mainly restricted to the subset expressing CD31 [platelet endothelial cell adhesion molecule (PECAM-1)] (14), a population that includes the RTEs and is thought to have a broadly diverse TCR repertoire (12). We have also shown that IL-7 increases the levels of expression of CD31 in this subset (14). The biological significance of CD31 expression is still debatable, though it has been suggested that it may limit TCR-mediated naive CD4 T-cell responses through inhibitory signaling ascribed to its cytoplasmic immune-receptor tyrosine-base inhibitory motifs (15). In agreement, the homeostatic proliferation of CD31^−^ naive CD4 T-cells is thought to be mainly mediated by low-affinity self-peptide/MHC interactions (16). Of note, CD31 expression is lost after TCR stimulation of naive CD4 T-cells (2, 17–19).

There are few studies on human naive CD4 T-cell homeostasis, and the interplay between peripheral mechanisms and the age-associated decline in thymic output remains unclear (3, 9, 10). Adults thymectomized early in infancy due to corrective cardiac surgery provide a unique setting to address this issue (20–27). Using this clinical model, we show here that thymic activity is required to ensure IL-7-mediated peripheral homeostatic proliferation, whereas the homeostasis of the CD31^−^ compartment is preserved in the absence of thymic activity.

## Materials and Methods

### Study Design

Blood was collected from 22 adult patients submitted to thymectomy during corrective cardiac surgery in early childhood and 20 age-matched healthy controls. All the subjects gave written informed consent for blood sampling and processing. The study was approved by the Ethical Boards of Faculdade de Medicina da Universidade de Lisboa, Centro Hospitalar Lisboa Norte, and Hospital de Santa Cruz, Portugal.

### Cell Isolation and Cell Culture

Peripheral blood mononuclear cells (PBMCs) were isolated from freshly collected heparinized blood *via* Ficoll-Paque PLUS (GE Healthcare, Uppsala, Sweden). Naive CD4 T-cells were subsequently purified by negative selection (purity > 96%, StemCell Technologies, Grenoble, France). Purified naive CD4 T-cells were cultured at 1 × 10^6^ cells/ml with either IL-7 (10 ng/ml; R&D Systems, Minneapolis, MN, USA) or IL-2 (20 IU/ml; NIH/AIDS Research and Reference Program, Division of AIDS, NIAID, Hoffman-La Roche), for up to 13 days (d), with media replacement at d3 and d7, as we have previously described (14, 28).

### Flow Cytometry

*Ex vivo* phenotypic analysis was performed in freshly collected whole blood using an eight-color staining protocol and a panel of monoclonal antibodies previously described (28). Purified naive CD4 T-cells were surface stained *ex vivo* and upon culture, followed by intracellular staining using eBioscience FoxP3 kit (eBioscience, San Diego, CA, USA), as described (28). At least 150,000 events were acquired for each sample on a BD LSRFortessa (BD Biosciences, San Jose, CA, USA). Data were analyzed using FlowJo software (TreeStar, Ashland, OR, USA) after doublet exclusion. Results are presented as proportion of a cell population or as mean fluorescence intensity (MFI) of a given marker within the specified population.

### TCR Activation

Purified untouched naive CD4 T-cells were cultured at 1 × 10^6^ cells/ml (25,000 cells/well) and stimulated with increasing concentrations of beads coated with anti-CD3 and anti-CD28 monoclonal antibodies (Dynabeads, Thermo Fischer Scientific), in order to generate a dose–response curve of TCR stimulation at the single-cell level. In agreement with a previously reported protocol (29), antibody against CD40L (clone 89-76, BD Bioscience) was added to the culture media to improve the staining. After 6 h, cells were collected and surface stained, as described (28), for the marker of cell differentiation (CD45RO) and the additional activation marker (CD69).

### TREC Quantification

Signal joint (sj) and DβJβTREC analyses were conducted as described (5, 30). Briefly, multiplex PCR amplification for sjTREC, DJβ1TRECs (Dβ1-Jβ1.1 to 1.6), or DJβ2TRECs (Dβ2-Jβ2.1 to 2.7), together with the CD3γ chain was performed in triplicate on lysed PBMC. TREC and CD3γ quantifications were then performed using a LightCycler™ in independent experiments, with the same first-round serial dilution standard curve. This highly sensitive nested quantitative PCR assay allowed detection of 1 copy in 10^5^ cells for any excision circle. The sj/βTREC ratio [sjTREC/10^5^ cells/(DJβ1TRECs/10^5^ cells + DJβ2TRECs/10^5^ cells)] was calculated as described (30).

### mRNA Quantification

Total RNA was extracted from purified naive CD4 T-cells using Quick-RNA MicroPrep (Zymo Research Corporation, Irvine, CA, USA). cDNA was synthesized from 50 ng of RNA (SuperScript III Reverse Transcriptase, Thermo Fischer Scientific) and used to quantify the expression levels of *KLF2, FOXP1, P21, BIM, DUSP4*, and *DUSP6* in duplicates, using TaqMan Gene Expression Assays on a ViiA7 Sequence Detection system (both from Thermo Fischer Scientific). Results are expressed as ΔCT normalized to the medium CT levels of *GAPDH* and *HPRT*.

### TCR Spectratyping Analysis

Total RNA was extracted from 10^5^ to 10^6^ cells with RNeasy kit (Qiagen, MD, USA), and first-strand cDNA synthesized from 1 to 2 µg of RNA (SuperScript III) using an equivolume mixture of random hexamers and oligo (dT). Amplification of the TCRVβ CDR3 was performed using primers specific for each TRBV family and a common TRCB reverse primer (31), followed by a run-off reaction that extends each different PCR product with a second TRCB FAM-labeled primer; and the third step, in which each different fluorescent TRBV-TRBC PCR fragment was separated using a capillary electrophoresis-based DNA automated sequencer. Data were collected and analyzed with GeneMapper v4.0 (Thermo Fischer Scientific) for size and fluorescence intensity determination.

### Statistical Analysis

Statistical analysis was performed with Graph Prism Version 5.01 (GraphPad Software, San Diego, CA, USA). The following tests were used for analyzing epidemiological data and results from *ex vivo* studies as appropriate: Wilcoxon-Signed Rank/paired *T*-test for pairwise comparisons and unpaired *T*-test/Mann–Whitney for unpaired comparisons, for Gaussian and non-Gaussian distribution respectively. Cultures were analyzed using one-way ANOVA. Results were expressed as median (interquartile range or range when *n* < 4). *P* values <0.05 were considered significant.

## Results

### Evidence of Thymus Activity in Adults Thymectomized during the First Year of Life

**Figure 1:**
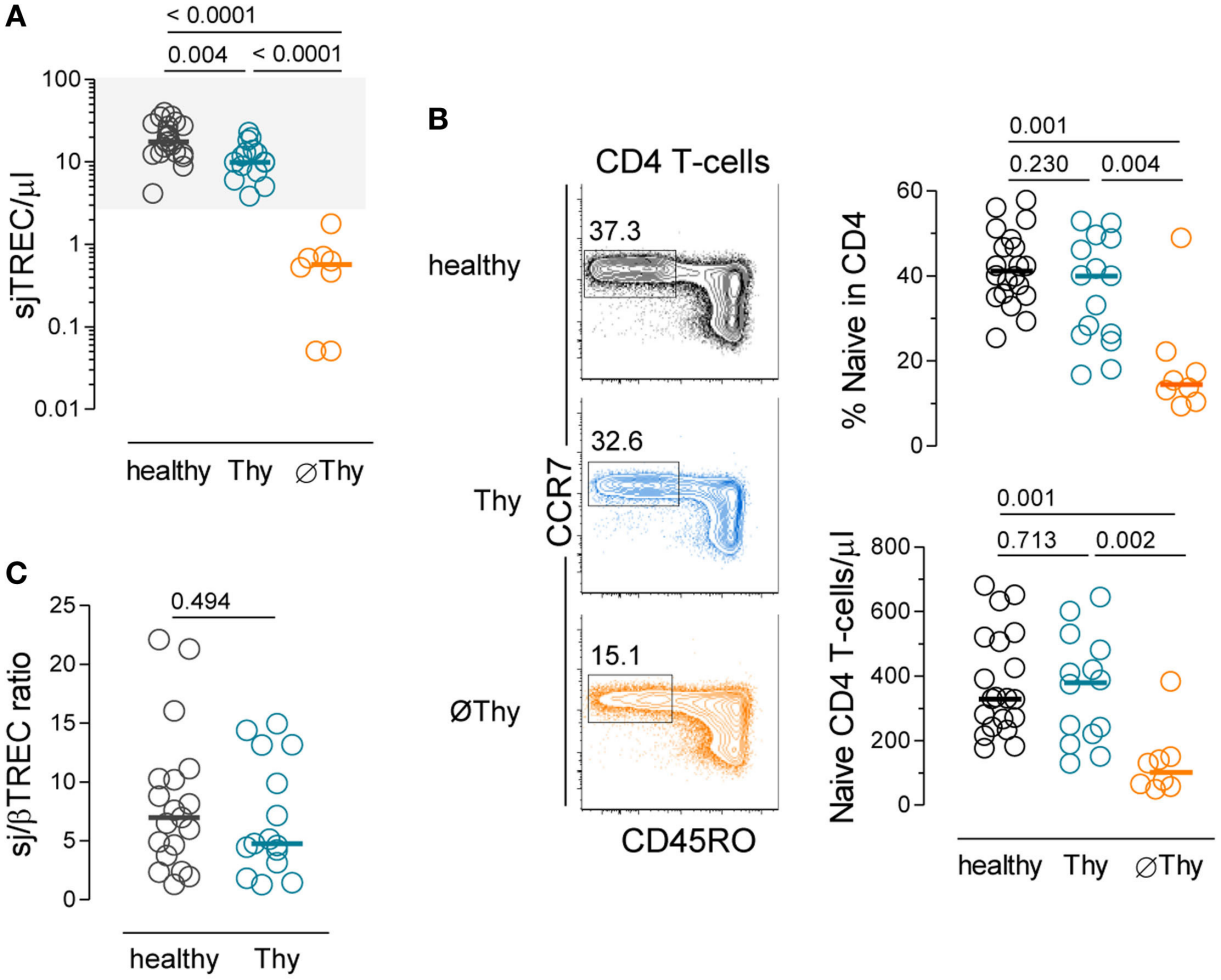
**Naive CD4 T-cell compartment in adults thymectomized early in life**. **(A)** Quantification of sjTREC levels in peripheral blood mononuclear cells (PBMCs) from thymectomized patients without (∅Thy), or with evidence of thymic activity (Thy), and in age-matched healthy individuals. **(B)** Contour plots illustrating CD45RO and CCR7 analysis within circulating CD4 T-cells of representative individuals from the three cohorts; graphs show naive CD4 T-cell frequency (top) and counts (bottom). **(C)** sj/βTREC ratio quantified in PBMCs from Thy and healthy individuals. Each dot represents one individual, bars represent median, and *P* values are shown.

We studied a cohort of 22 adults submitted to thymectomy in early childhood during corrective cardiac surgery and 20 age-matched healthy controls (Table 1; Table S1 in Supplementary Material). Of note, the thymic function is relatively stable in healthy individuals during the age-period spanned (4). The thymectomized patients were stratified into two groups according to evidence of residual thymic activity (Table 1; Table S1 in Supplementary Material). No thymic activity (∅Thy) strictly refers to cases with surgical reports of complete thymus removal and levels of sjTRECs clearly below the lower level found in controls (*P* < 0.0001, Figure 1A), as we previously reported (28). Individuals with some degree of thymic activity (Thy) featured sjTREC levels within the range of age-matched controls, though significantly lower (*P* = 0.0061, Figure 1A).

**Table 1:** **Clinical–epidemiological characteristics of cohorts**.

	Healthy	Thy^a^	ØThy^a^
Number (male/female)	20 (8/12)	14 (6/8)	8 (5/3)
Age, years	22 [18–29]	25 [18–30]	23 [20–27]
Age at thymectomy, months	NA	8 [1–60]	21 [12–72]
Total lymphocytes/μl	2,408 [1,430–3,502]	2,219 [1,230–3,400]	2,005 [934–2,618]*
% T-cells (CD3^+^)	72.7 [50.2–79.0]	70.5 [57.3–82.7]	66.6 [42.7–71.2]*^,#^
% CD4 T-cells	39.9 [31.2–60.0]	41.0 [33.8–55.3]	42.7 [20.20–46.4]
Serum IL-7^b^, pg/ml	15.0 [6.5–23.3]	12.8 [5.3–16.2]*	14.8 [8.3–19.8]

*NA indicates not applicable. Results are shown as median and range in brackets*.

*P-value <0.05 in comparison with healthy.

*^#^P-value <0.05 in comparison with Thy*.

*^a^Thymectomy was performed during reconstructive cardiac surgery to facilitate surgical access to the heart and great vessels; patients with syndromatic cardiopathy were excluded (e.g., trisomy 21, velocardiofacial syndrome, or DiGeorge syndrome); individuals were not treated with drugs known to influence the immune system; ØThy, no thymic activity based on surgical reports of complete thymus removal and levels of sjTRECs clearly below the lower level found in controls; Thy, some degree of thymic activity attested by sjTREC levels within the range of age-matched controls; none of the individuals featured increased rate of infections or autoimmune manifestation*.

*^b^Serum IL-7 levels were quantified using Human IL-7 Quantikine HS ELISA kit (R&D Systems)*.

Interestingly, these two groups showed almost no overlap between age at thymectomy, which was performed during the first year of life in all Thy cases except two and later on in all ∅Thy patients (Table S1 in Supplementary Material). A previous study also found an association between preservation of thymic activity and younger age at thymectomy, which was attributed to loss of thymus regenerative capacity in children older than 18 months (24, 32). It is also noteworthy that complete thymic tissue removal is more likely to occur after the first year of life due to the surgical procedures required for the type of cardiac defects (Table S1 in Supplementary Material), as well as due to age-related anatomic specificities (21) Of note, individuals with syndromatic cardiac defects were not included (Table S1 in Supplementary Material).

In agreement with complete lack of thymic activity, the ∅Thy group featured decreases in naive CD4 T-cell frequency and absolute numbers, which were statistically significant not only in comparison with healthy individuals (*P* = 0.0012 and *P* = 0.0006, respectively, Figure 1B) but also with Thy (*P* = 0.0041 and *P* = 0.0019, respectively, Figure 1B). These cells featured a truly naive phenotype based on an extensive panel of naive markers and lack of expression of molecules associated with a memory phenotype, as we have previously reported (28).

On the other hand, Thy patients showed no reduction in lymphocyte counts (Table 1) and maintained the naive CD4 T-cell compartment (Figure 1B). In order to estimate their effective thymic output, we quantified the sj/βTREC ratio, which reflects the number of proliferation cycles undergone by precursor T-cells during their intra-thymic differentiation and directly correlates with thymic activity (30). We observed similar levels of sj/βTREC ratio in Thy and age-matched healthy individuals (Figure 1C). Together with close to normal sjTREC contents, this observation supports a major contribution of thymic recovery to the maintenance of the size of naive CD4 T-cell compartment upon partial thymectomy.

**Figure 2:**
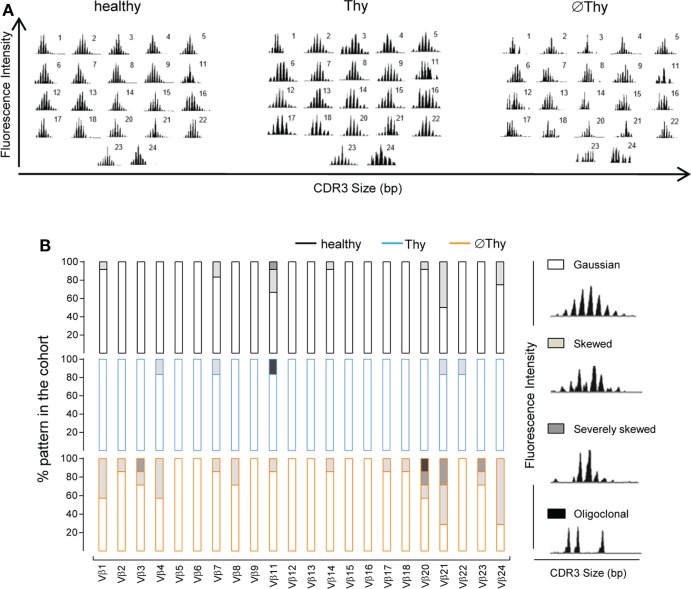
**Spectratyping analysis of naive CD4 T-cell diversity in adults thymectomized early in life**. CD3 length distribution within each of the 22 Vβ families of purified naive CD4 T-cells analyzed by spectratyping: **(A)** illustrative examples from thymectomized patients without (right, ∅Thy1 in Table S1 in Supplementary Material) and with evidence of thymic activity (middle, Thy6 in Table S1 in Supplementary Material), as well as age-matched healthy (left) individuals; **(B)** proportion of the illustrated patterns within each Vβ family in the three cohorts (∅Thy, bottom, *n* = 7; Thy, middle, *n* = 6; healthy, top, *n* = 12).

We further assessed the impact of the degree of thymectomy on the structural diversity of naive CD4 T-cells by spectratyping analysis of their TCR repertoire. The distribution of the CDR3 lengths within each different Vβ family is considered to reflect the overall sequence diversity (33). A diverse polyclonal TCR repertoire is associated with a Gaussian distribution of CDR3 lengths, whereas skewed TCR repertoires feature a reduced number of peaks. A relatively preserved TCR diversity was observed in Thy patients as compared to age-matched controls (*P* = 0.2620), supporting that their degree of thymic activity was sufficient to ensure the preservation of the quality of the naive CD4 T-cell compartment (Figures 2A,B). Patients with no thymic activity exhibited higher numbers of non-polyclonal Gaussian families than both Thy (*P* < 0.0001) and healthy individuals (*P* = 0.0001), as shown in Figure 2.

Of note, these differences between the two thymectomized cohorts could not be attributed to a distinct prevalence of CMV infection, since a similar proportion of individuals with IgG seropositivity against CMV was observed in ∅Thy and Thy cohorts (Table S1 in Supplementary Material). Moreover, the contraction of the naive CD4 T-cell compartment in ∅Thy individuals was not restricted to those seropositive for CMV, as previously reported (21).

Overall, peripheral homeostatic mechanisms were unable to prevent the contraction of the naive CD4 T-cell compartment upon complete thymus removal in infancy, whereas the maintenance of some degree of thymic activity allowed preservation of naive CD4 T-cells with a diverse TCR repertoire into adulthood.

### Lack of Thymic Activity Does Not Associate with Increased Threshold for TCR Activation of Naive CD4 T-Cells

**Figure 3:**
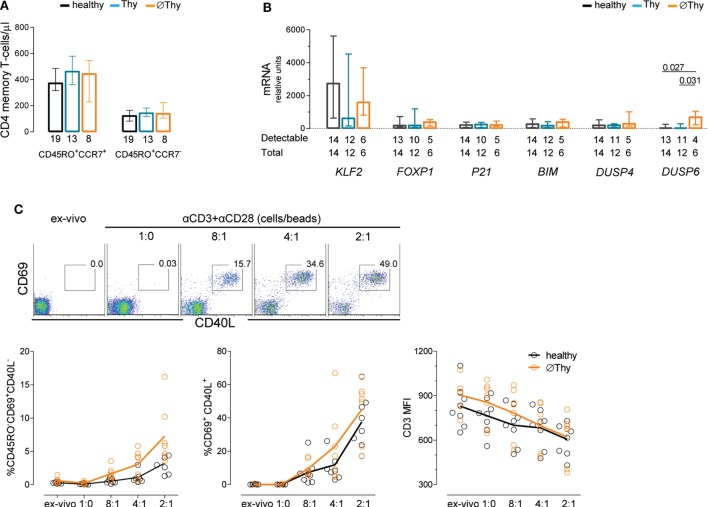
**Maintenance of naive CD4 T-cell quiescence upon thymectomy**. **(A)** Absolute numbers of circulating central memory (CD45RO^+^CCR7^+^) and effector memory (CD45RO^+^CCR7^−^) cells in thymectomized patients without (∅Thy) and with evidence of thymic activity (Thy), as well as age-matched healthy individuals; numbers below the graph indicate the number of individuals. **(B)** mRNA expression levels of genes involved in cell cycle or maintenance of naive phenotype quantified in purified naive CD4 T-cells from the three cohorts; ΔCT normalized to the medium CT levels of *GAPDH* and *HPRT* are shown; numbers below the graph indicate the total number of samples tested and those with levels above the detection threshold of the respective gene. **(C)** Purified naive CD4 T-cells from ∅Thy and healthy individuals were stimulated (6 h) with increasing concentrations of beads coated with anti-CD3 and anti-CD28 monoclonal antibodies with dot plots illustrating the upregulation of the activation markers CD40L and CD69 in one ∅Thy subject, and graphs showing frequencies of CD69^+^CD40L^−^ cells (left), CD69^+^CD40L^+^ cells (middle), and CD3 mean fluorescence intensity (right); each dot represents one individual; lines connect means; and the two cohorts were compared with two-way ANOVA. Bars represent median and interquartile range. *P* values <0.05 are shown.

The maintenance of naive T-cells is also determined by the rate of their differentiation into memory–effector cells. Of note, both central and effector memory CD4 T-cell counts in individuals lacking thymic activity were found to be similar to those in age-matched healthy controls (Figure 3A). Therefore, we assessed the expression of a panel of genes known to be involved in the regulation of cell quiescence and/or of the threshold for TCR-mediated cell activation in purified naive CD4 T-cells from thymectomized and healthy individuals (Figure 3B). No alterations were found in the expression levels of the following genes: Krüppel-like factor 2 (*KLF-2*) (34), the transcription factor *FOXP1* (35), *CDKN1A* (encoding the cyclin-dependent kinase inhibitor p21^cip1/waf^) (36), the proapoptotic Bcl-2 family member *BIM* (37), and the dual-specificity protein phosphatase *DUSP4* (38). However, individuals with no thymic activity featured significantly higher *DUSP6* transcript levels than controls, an increase not observed in those with some preservation of thymopoiesis (Figure 3B).

DUSP6 is highly specific for ERKs, leading to reduction of ERK activity that is critical for efficient TCR signaling (38, 39). Therefore, high *DUSP6* levels might be associated with an increase in the threshold for TCR-induced activation in ∅Thy. To test this possibility, we performed a dose–response TCR stimulation of purified naive CD4 T-cells and quantified the upregulation of the early activation markers CD40L and CD69, in parallel with the downregulation of CD3 expression and induction of the memory marker CD45RO. Contrarily to our expectation, individuals completely lacking thymic activity responded to TCR stimulation as efficiently as healthy subjects (Figure 3C).

In conclusion, we found no apparent constraint in the differentiation of naive CD4 T-cells into the memory compartment in individuals completely lacking thymic activity.

### Preservation of the CD31^−^ Compartment of CD4 Naive T-Cells in the Absence of Thymic Activity

The CD31 molecule has been shown to be expressed in all RTEs and to be lost upon TCR stimulation of naive CD4 T-cells (12, 18). Next, we investigated the contribution of peripheral cell survival and cell cycling to the homeostasis of the CD31^+^ and CD31^−^ naive CD4 T-cell subsets according to the degree of thymic activity.

**Figure 4:**
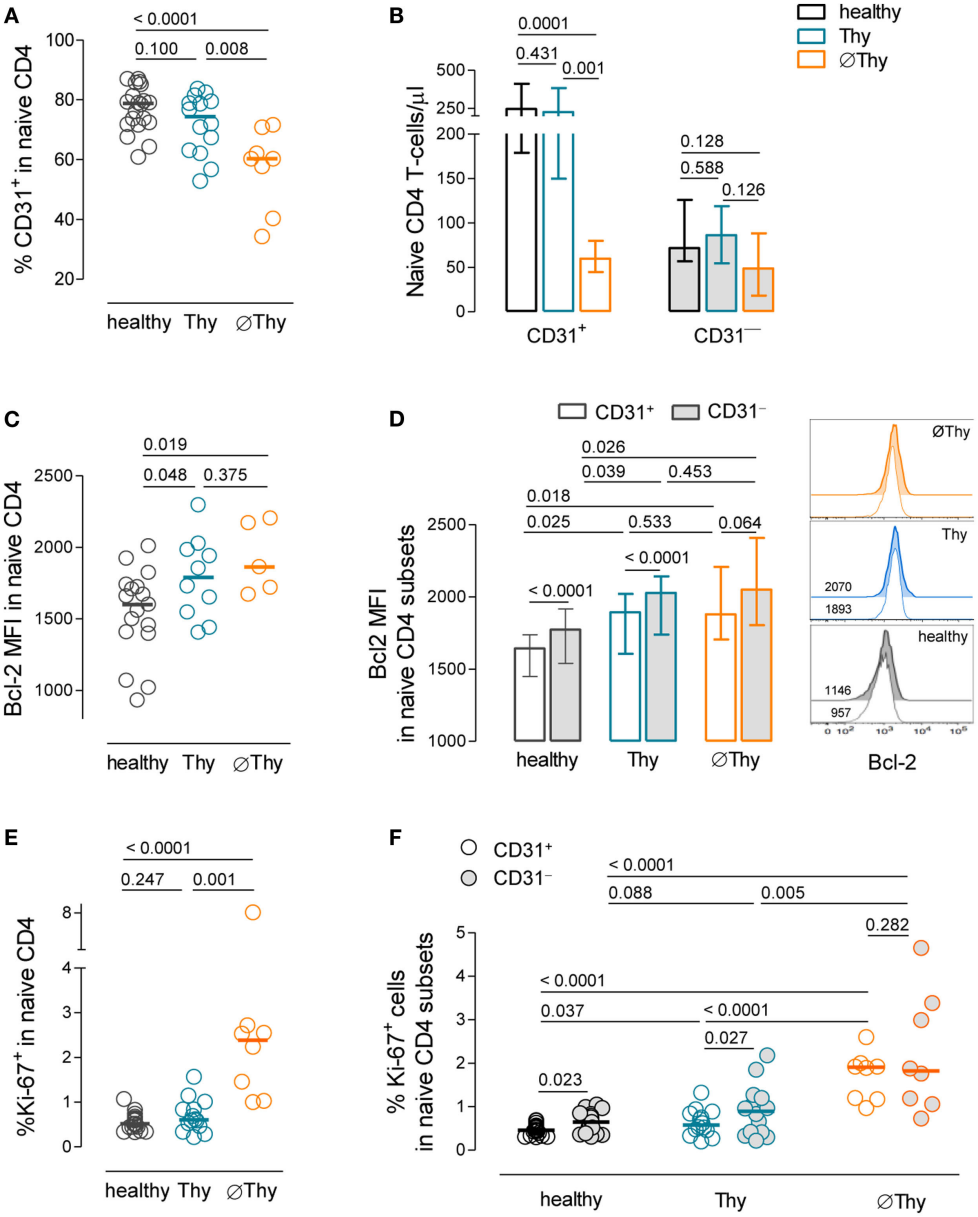
**Homeostasis of the CD31^+^ and CD31^−^ subsets of naive CD4 T-cells upon thymectomy**. **(A)** Frequency of CD31^+^ cells within naive CD4 T-cells in thymectomized patients without (∅Thy) and with evidence of thymic activity (Thy), as well as age-matched healthy individuals. **(B)** Absolute numbers of CD31^+^ and CD31^−^ naive CD4 T-cells in ∅Thy (*n* = 8), Thy (*n* = 14), and healthy (*n* = 19) individuals. **(C)** Bcl-2 mean fluorescence intensity (MFI) within naive CD4 T-cells. **(D)** Bcl-2 MFI within the CD31^+^ and CD31^−^ subsets in ∅Thy (*n* = 5), Thy (*n* = 9), and healthy (*n* = 17) individuals, as illustrated in the representative overlay histograms (numbers indicate Bcl-2 MFI). **(E,F)** Frequency of cycling cells (Ki-67^+^) within naive CD4 T-cells **(E)** and within the CD31^+^ and CD31^−^ subsets **(F)** in the three cohorts. In scatter graphs, each dot represents one individual, and bars represent median; bar graphs show median and interquartile range. *P* values are shown.

The CD31^+^ compartment was preserved in Thy patients (Figures 4A,B). Conversely, it was significantly contracted in the ∅Thy cohort, both in frequency (Figure 4A) and absolute counts (Figure 4B), as expected in the absence of thymic activity (3, 20). Of note, the median level of CD31 expression within CD31^+^ naive CD4 T-cells was not significantly different in thymectomized individuals (CD31 MFI: healthy 4,494 [3,490–4,872]; Thy 3,573 [3,255–4,453]; ØThy 3,936 [2,658–4,170]; *P* > 0.05).

Importantly, despite the marked naive CD4 T-cell lymphopenia, ∅Thy featured preserved CD31^−^ naive CD4 T-cell counts (Figure 4B). This finding adds to previous data on aged individuals reporting preservation of the CD31^−^ compartment in parallel with the progressive decline of CD31^+^ cell counts (40) and argues in favor of the robustness of the homeostasis of CD31^−^ naive CD4 T-cells in individuals lacking thymic activity.

The expression levels of the survival marker Bcl-2 were upregulated in both thymectomized cohorts as compared to healthy controls (∅Thy: *P* = 0.0187; Thy: *P* = 0.0487; Figure 4C), suggesting increased naive CD4 T-cell survival irrespectively of the presence of thymic activity, which persisted for more than 20 years post-thymectomy (Table 1). This increase was observed in both CD31^+^ and CD31^−^ naive T-cells (Figure 4D). Of note, we found that the CD31^−^ compartment featured significantly higher Bcl-2 MFI than CD31^+^ cells both in healthy and Thy individuals, a difference that was attenuated in ∅Thy (Figure 4D).

Regarding proliferation, a significant increase in the frequency of cycling cells within total naive CD4 T-cells was found only in ∅Thy (*P* < 0.0001 to healthy, *P* = 0.0007 to Thy; Figure 4E). The proportion of Ki-67^+^ cells was significantly higher in the CD31^−^ than in the CD31^+^ compartment in both healthy individuals and in Thy patients (Figure 4F). Nevertheless, its relative contribution to the pool of proliferating naive CD4 T-cells is minor (<1%), given the large overrepresentation of CD31^+^ cells in these individuals (Figure 4A). On the other hand, ∅Thy individuals featured an increase in the relative representation of the CD31^−^ subset, associated with the loss of CD31^+^ cells (Figure 4A), in parallel with a significant increase in the frequency of cycling cells irrespective of CD31 expression (CD31^−^ subset: *P* < 0.0001 to healthy; *P* = 0.0048 to Thy; CD31^+^ subset: *P* < 0.0001 to healthy; *P* < 0.0001 to Thy; Figure 4F).

Thus, we showed that the CD31^−^ naive CD4 T-cell compartment was maintained in the absence of thymic output, in association with both expanded cell survival and increased proliferation.

### Naive CD4 T-Cells Feature Reduced Proliferative Response to IL-7 *In Vitro* in the Absence of Thymic Activity *In Vivo*

**Figure 5:**
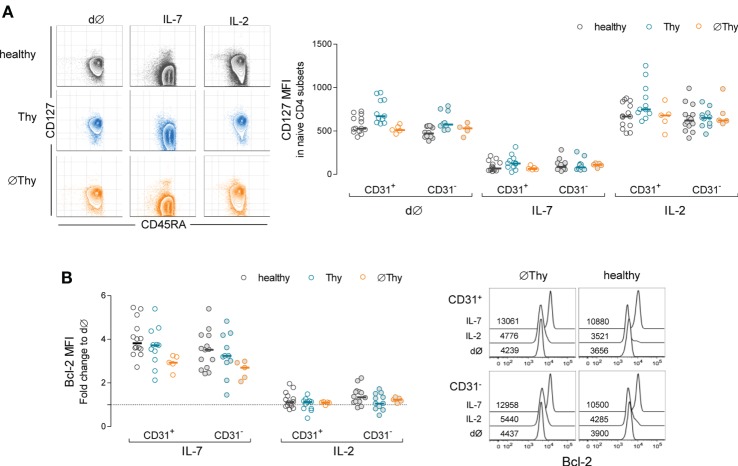
**Impact of thymectomy on naive CD4 T-cell ability to respond to IL-7**. Purified naive CD4 T-cells from thymectomized individuals without (∅Thy) or with evidence of thymic activity (Thy), and age-matched healthy controls were cultured for 13d with IL-7 or IL-2. **(A)** Illustrative contour plots from one individual of each cohort showing maintenance of naive phenotype (CD45RA^+^) and downregulation of IL-7Rα (CD127) upon culture with IL-7 but not with IL-2; graph shows CD127 mean fluorescence intensity (MFI) within the CD31^+^ and CD31^−^ compartments *ex vivo* (d∅) and upon culture with IL-2 or IL-7 in the three cohorts: CD127 downregulation in response to IL-7 was statistically significant as compared to both d∅ and IL-2 in all cohorts/subsets (*P* < 0.001); no significant differences were found between d∅ and IL-2, except for CD31^+^ cells in healthy (*P* < 0.05); the comparison between cohorts revealed no significant differences, except for the levels of CD127 MFI at d∅ in Thy in comparison to both healthy (CD31^+^: *P* < 0.01; CD31^−^: *P* < 0.001) and ∅Thy (CD31^+^: *P* < 0.01) individuals. **(B)** Fold change of Bcl-2 MFI within CD31^+^ and CD31^−^ naive CD4 T-cells upon culture with IL-7 or IL-2 as compared to d∅ in the three cohorts: Bcl-2 upregulation with IL-7 was significant in all cohorts/subsets (*P* < 0.001) without inter-cohort differences, except for CD31^+^ naive CD4 T-cells in ∅Thy in comparison with healthy (*P* < 0.05); overlay histograms illustrate Bcl-2 expression within gated CD31^+^ and CD31^−^ naive CD4 T-cells in d∅ and upon culture with IL-7 or IL-2 in a ∅Thy and an healthy individual. Each dot represents one individual, and bars represent median.

Cytokine-driven homeostatic mechanisms are crucial for naive CD4 T-cell maintenance, and IL-7 is considered the key cytokine in these processes (13). Therefore, we hypothesized that naive CD4 T-cells adjust their intrinsic ability to respond to IL-7 in order to counteract the decline in thymic output. Thus, we investigated the impact of IL-7 on purified naive CD4 T-cells, using a 13d culture system previously optimized in our laboratory (14, 28). We were able to purify the required amount of naive CD4 T-cells from 5 ØThy and 11 Thy individuals, which were compared with samples from 14 healthy subjects. We found comparable *ex vivo* levels of IL-7Rα-chain (CD127) expression within naive CD4 T-cells in healthy and ∅Thy, and significantly higher levels in Thy individuals (Figure 5A). Others have shown that the proximal signaling through the IL-7 receptor is preserved in adults thymectomized in infancy, as assessed by STAT5 phosphorylation upon short-term stimulation with IL-7 (20).

As illustrated in Figure 5A, the cells preserved their naive phenotype upon culture with either IL-7 or IL-2, including those from ∅Thy individuals. The expected IL-7-mediated downregulation of CD127 expression (14, 41) was comparable in all individuals and, therefore, independent of the degree of thymic activity (Figure 5A). Of note, no changes occurred in the control culture condition with IL-2 (Figure 5A). Additionally, the upregulation of CD25 and CD95 by IL-7 (14, 42) was also similar in all cohorts, both in the CD31^+^ and CD31^−^ compartments (data not shown).

An important physiological role of IL-7 relies on Bcl-2 induction (43). We found a clear upregulation of Bcl-2 expression, in both CD31^+^ and CD31^−^ subsets in cultures with IL-7 in both thymectomized cohorts, which was not observed with IL-2 (Figure 5B).

**Figure 6:**
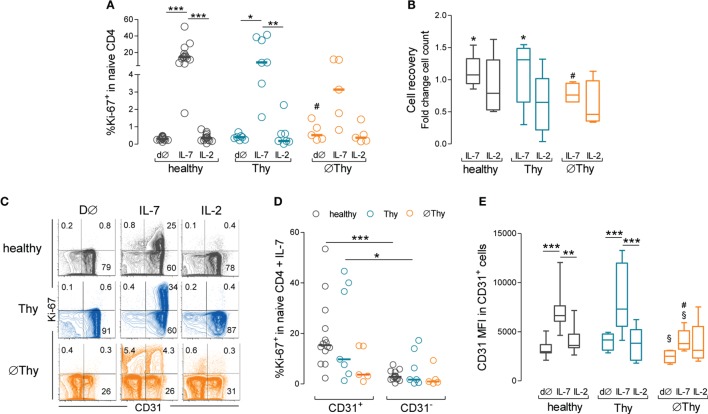
**Impact of thymectomy on naive CD4 T-cell ability to proliferate *in vitro* in response to IL-7**. Purified naive CD4 T-cells from thymectomized patients without (∅Thy) or with evidence of thymic activity (Thy), and from age-matched healthy controls were cultured for 13d with IL-7 or IL-2. **(A)** Frequency of cycling cells (Ki-67^+^) within total naive CD4 T-cells *ex vivo* (d∅) and upon culture with IL-7 or IL-2. **(B)** Cell recovery counts upon culture with IL-7 or IL-2. **(C)** Contour plots illustrate the analysis of Ki-67 versus CD31 in representative ∅Thy, Thy, and healthy individuals. **(D)** Frequency of Ki-67^+^ cells within CD31^+^ and CD31^−^ naive CD4 T cells. **(E)** CD31 expression (mean fluorescence intensity) within the CD31^+^ compartment at d∅ and upon culture with IL-7 or IL-2 in ∅Thy (*n* = 5), Thy (*n* = 7), and healthy (*n* = 14) individuals. Each dot represents one individual, and bars represent median and interquartile range. Significant *P* values are shown: *P < 0.05, **P < 0.01, and ***P < 0.001 for comparisons between conditions within each cohort; ^#^*P* < 0.05 for comparisons between healthy and ∅Thy or Thy; ^§^*P* < 0.05 for comparisons between ∅Thy and Thy.

We then investigated the proliferative response of naive CD4 T-cells using the cell-cycling marker Ki-67, which we have shown to be the best approach to reveal low-level IL-7-driven proliferation (14). Unexpectedly, we found that in contrast to healthy and Thy cohorts, ∅Thy featured no significant increase in the frequency of cycling cells in response to IL-7, as compared to both *ex vivo* and cultures with IL-2 (Figure 6A). As shown in Figure 6B, naive CD4 T-cell recovery per well was significantly higher upon culture with IL-7 than IL-2 in healthy (fold change 1.07 [0.86–1.54] versus 0.79 [0.51–1.63]; *P* = 0.0137) and Thy (fold change 1.15 [0.16–1.55] versus 0.65 [0.06–1.32]; *P* = 0.0259), but not in ∅Thy (fold change 0.87 [0.65–1.77] versus 0.54 [0.34–1.66]; *P* = 0.2234). The cell recovery upon culture with IL-7 was significantly lower in ∅Thy as compared to healthy individuals (*P* = 0.0236).

We have previously shown that the IL-7-induced proliferation was restricted to the CD31^+^ subset in healthy subjects (14), which was confirmed here (Figures 6C,D). Notably, a similar profile was observed in Thy individuals (Figures 6C,D). By contrast, patients lacking thymic activity featured reduced proliferation upon IL-7 stimulation (Figures 6C,D) and showed no significant increase in the frequency of cycling cells (Ki-67^+^) within the CD31^+^, as compared to the CD31^−^ subset (Figures 6C,D). As a consequence, the proportion of CD31^+^ within cycling cells was significantly lower in ∅Thy, as compared to both healthy and Thy individuals (*P* < 0.0001 and *P* = 0.007, respectively).

We have also previously shown that IL-7 upregulates the levels of expression of CD31 within the CD31^+^ naive CD4 T-cell subset in a PI3K-dependent manner (14, 28). Of note, the ∅Thy cohort reached significantly lower levels of upregulation of CD31 MFI within the CD31^+^ subset in response to IL-7, as compared to healthy (*P* = 0.0035), despite featuring comparable *ex vivo* levels (*P* = 0.1052) (Figure 6E). Moreover, the upregulation of CD31 MFI was also significantly lower in ∅Thy than in Thy (*P* = 0.0177) individuals, although the latter featured significantly higher *ex vivo* levels of CD31 MFI within the CD31^+^ subset (*P* = 0.0177) (Figure 6E). These findings suggest that naive CD4 T-cells from ∅Thy patients lost the ability to respond to IL-7, possibly through the PI3K pathway, in agreement with their impaired proliferation. Conversely, they preserved the ability to upregulate Bcl-2 in response to IL-7, which we have shown previously that is not PI3K-dependent (14, 28).

Altogether, these data showed that the ability of naive CD4 T-cells to proliferate and upregulate CD31 in response to IL-7 was impaired in individuals completely lacking thymic activity.

## Discussion

We investigated here mechanisms of peripheral naive CD4 T-cell homeostasis in adults with different degrees of thymus impairment since early infancy. We found that the size of the CD31^−^ compartment was similar in healthy and thymectomized subjects, supporting the existence of thymus-independent homeostasis, possibly driven by self-peptide/MHC. On the other hand, proliferation mediated by IL-7, the main homeostatic cytokine, was severely impaired in the absence of thymopoiesis.

Thymectomy performed during corrective cardiac surgery in infancy is widely recognized as a powerful model to investigate the thymus contribution to naive T-cell maintenance beyond the establishment of the T-cell compartment. Nevertheless, a wide heterogeneity of findings has been reported (22–26, 32, 44–47). Our study focused on adults thymectomized during infancy/early childhood within a relatively narrow age range, which were grouped according to absence (∅Thy) or presence (Thy) of thymopoiesis based on circulating sjTRECs/μl (5). Our molecular strategy to stringently rule out the existence of thymic output in thymectomized patients overcomes the limitations of other approaches based solely on surgical reports (21, 23, 32, 44–46) and/or thoracic imaging (21, 32, 45, 48), which may have neglected thymic regeneration or ectopic thymus (49).

Of note, after the exclusion of the thymectomized patients lacking thymic activity, we found that both size and diversity of the naive CD4 T-cell compartment were preserved to a median of 21 years post-thymectomy. This likely occurred through both peripheral mechanisms and thymus regeneration, as supported by our finding of sj/βTREC ratios in Thy patients within the range of healthy age-matched controls. These data strengthen the recommendation to avoid complete thymectomy during cardiac surgery (44, 50), which is particularly relevant after the first year of life given the observed association between younger age at thymectomy and thymic recovery (24, 32).

The thymus provides a unique environment to generate a diverse TCR repertoire (51). This process that involves genomic recombination and gene editing at the individual cell level (52) imposes major challenges to the quantification of TCR diversity, particularly when sample availability is limited (52–57), leading us to opt for a standard approach using spectratyping. To our knowledge, there is only one study assessing the diversity of purified naive CD4 T-cells from three thymectomized children/adolescents that reported conservation of the spectratyping profiles (25). We showed here that the diversity of the TCR repertoire within the naive CD4 T-cell compartment was preserved in thymectomized individuals with some degree of remaining thymic activity and significantly contracted in patients completely lacking thymopoiesis. Although ØThy featured no major infections or autoimmunity, this profile of premature immune senescence (21, 22, 46, 58) is likely to have clinical implications not yet evaluated, since successful corrective cardiac surgery in young children only became a routine practice three decades ago, precluding extended follow-up studies (45, 59).

Of note, patients lacking thymic activity featured no major change of the transcript levels of genes involved in cell quiescence and survival of naive CD4 T-cells, except for the significant increase in *DUSP6*. This phosphatase enhances the TCR activation threshold by decreasing ERK phosphorylation (39). However, no significant change was observed in the activation of purified naive CD4 T-cells, suggesting that this pathway does not limit their differentiation into the memory–effector pool in completely thymectomized patients. Accordingly, they featured an increase in cycling cells within the CD31^−^ subset that is thought to mainly proliferate in response to TCR stimulation by low-affinity self-peptide/MHC (2, 18).

We showed here that the maintenance of the CD31^−^ subset is independent of thymic output, and that robust peripheral mechanisms ensure the homeostasis of this population. This is in agreement with the CD31^−^ preservation that others have reported during age-associated thymic involution (12, 40). Our study revealed that, even in healthy young adults, the levels of the prosurvival molecule Bcl-2 were significantly higher in CD31^−^ than in CD31^+^ naive CD4 T-cells, emphasizing the contribution of anti-apoptotic pathways for the homeostasis of the CD31^−^ subset (60).

IL-7 is known to play a crucial role in naive CD4 T-cell homeostasis, not only by enhancing thymopoiesis (61, 62) but also through the peripheral induction of survival and proliferation (14, 28, 56, 63). We show here that there is no reduction of IL-7Rα expression within naive CD4 T-cells from thymectomized individuals, and others have shown that IL-7Rα proximal signaling, as assessed by STAT5 phosphorylation, were preserved (20). Our data support the notion that in the absence of thymopoiesis, there is mainly an impairment in the peripheral responses to IL-7 that are PI3K-dependent, namely, proliferation and CD31 upregulation, whereas Bcl-2 induction, which does not rely on this pathway, is relatively preserved. We have previously reported preservation of naive regulatory T-cells in the same ØThy cohort, despite the marked contraction of conventional naive CD4 T-cells (28). In the current study, we further analyzed the expression of the regulatory marker FoxP3 within cycling naive CD4 T-cells, and found that in contrast to the conventional cells, the FoxP3^+^ featured significant proliferation rates upon culture with IL-7 (median fold change of Ki-67^+^ cells as compared to *ex vivo* levels: 3.41 for FoxP3^+^ versus 1.32 for Foxp3^−^, *P* = 0.0248, *n* = 5). These findings point to a defect of conventional naive CD4 T-cells. The comparison of the two naive CD4 T-cell subsets is therefore a promising strategy to clarify the mechanisms underlying the defective IL-7 response in complete thymectomized individuals. These data will foster our understanding of IL-7 signaling in human naive CD4 T-cells and possibly identify druggable targets.

Functional heterogeneity within naive CD4 T-cells may result from the maturation process that RTEs undergo in the periphery, which may vary throughout life (64–66). It is expectable that cells with privileged response to IL-7 are more abundant in the first years of life, when accelerated growth and constant exposure to new antigens demand for peripheral expansion to ensure continuous replenishment of the naive compartment. IL-7-induced proliferation is known to be higher in mature single-positive thymocytes than peripheral T-cells (28, 66) and in cord blood than adult naive CD4 T-cells (14). Moreover, it is plausible that in elderly, an impaired ability of circulating naive CD4 T-cells to proliferate in response to IL-7 contributes to their decline, in parallel with thymic involution (40). In this context, the low-level homeostatic proliferation of naive CD4 T-cells will progressively rely on self-peptide MHC interactions, which in addition to constrain the repertoire may promote aging-associated autoreactivity.

Our data suggest a scenario where proliferative responses to IL-7 would be favored in a narrow window of time upon thymic egress, which has important implications to the therapeutic use of IL-7 in clinical settings known to be associated with thymic injury, namely, HIV/AIDS (67, 68) and chemotherapy (69, 70). Therefore, the requirement for ongoing thymopoiesis questions the suggested benefit of IL-7 therapy in the recovery of lymphopenia in thymectomized individuals (21).

In complete thymectomized individuals, the homeostatic proliferation of naive CD4 T-cells is likely to be mostly related to TCR stimulation by low-affinity self-peptide/MHC in both CD31^−^ and CD31^+^ subsets, which likely contributes to further constrain their TCR repertoire.

In conclusion, our investigation of the interplay of thymic output and peripheral mechanisms to the maintenance of the naive CD4 T-cell compartment uncovered the need for continued thymic activity to the IL-7-driven peripheral proliferation of naive CD4 T-cells. These findings are of particular relevance for lymphopenic clinical settings and aging, demanding the appraisal of thymus targeting strategies in order to maximize the peripheral effect of IL-7.

## Ethics Statement

The study was approved by the Ethical Boards of Faculdade de Medicina da Universidade de Lisboa, Centro Hospitalar Lisboa Norte, and Hospital de Santa Cruz, Portugal. All the subjects gave written informed consent for blood sampling and processing. Vulnerable populations, namely, minors, pregnant women, or persons with disabilities were not included.

## Author Contributions

SS, AA, JB, RV, and AS designed the study; SS, AA, PM, BC-M, DL, and RC performed research; SS, MA, and RA collected clinical data; AS supervised the study; SS and AS wrote the paper.

## Conflict of Interest Statement

The authors declare that the research was conducted in the absence of any commercial or financial relationships that could be construed as a potential conflict of interest.
