# Supplementary material for: IL-7-Induced Proliferation of Human Naive CD4 T-Cells Relies on Continued Thymic Activity
Source: Front Immunol. 2017 Jan 19;8:20. doi: 10.3389/fimmu.2017.00020 (PMC5243809; doi:10.3389/fimmu.2017.00020)
Supplement: Supplementary file 1 [file Table_1.PDF]

Supplemental Table 1. Epidemiological, clinical and immunological characteristics of thymectomized individuals

Patient	Age (years) / gender	Cardiac defect	Age at thymectomy (months)	Surgical report of thymus removal	sjTREC/µl	Naive CD4+ T-cells		CMV[a] (IgG)
						% within total CD4	cells/µl	
Thy 1	21/M	Tricuspid atresia	10	partial	12.83	48.7	407.5	+
Thy 2	24/M	Coarctation of the aorta	3	partial	7.53	49.5	478.9	–
Thy 3	26/F	Anomalous pulmonary venous return	11	partial	8.85	17.9	149.5	+
Thy 4	28/F	Transposition Great Arteries	11	partial	22.64	41.6	529.2	–
Thy 5	20/F	Transposition Great Arteries	1	partial	9.90	24.5	239.8	+
Thy 6	19/F	Ventricular septal defect	8	partial	11.52	28.2	187.0	+
Thy 7	18/M	Ventricular and atrial septal defects	8	partial	12.52	26.3	418.4	+
Thy 8	26/F	Double outlet right ventricle	60	complete	4.97	26.0	218.6	+
Thy 9	25/M	Transposition Great Arteries + pulmonary stenosis	15	complete	9.81	46.0	373.1	–
Thy 10	28/F	Transposition Great Arteries	7	partial	13.76	37.0	599.1	+
Thy 11	29/F	Transposition Great Arteries + ventricular septal defect	3	partial	3.80	16.6	127.1	–
Thy 12	30/M	Transposition Great Arteries	3	partial	9.68	33.0	245.7	+
Thy 13	27/F	Transposition Great Arteries	8	partial	19.45	39.9	385.9	–
Thy 14	17/M	Anomalous pulmonary venous return	7	partial	18.18	52.8	642.6	+
∅Thy 1	20/F	Tetralogy of Fallot	18	complete	0.62	13.6	63.7	+
∅Thy 2	22/F	Tetralogy of Fallot	23	complete	0.52	13.4	138.5	+
∅Thy 3	24/M	Tetralogy of Fallot	72	complete	0.05	48.8	381.9	–
∅Thy 4	22/F	Tetralogy of Fallot	48	complete	1.76	15.3	128.6	–
∅Thy 5	22/M	Pulmonary atresia	12	complete	0.71	22.1	56.2	+
∅Thy 6	26/M	Tetralogy of Fallot	48	complete	0.45	17.2	148.1	+
∅Thy 7	27/M	Tetralogy of Fallot	12	complete	0.67	10.3	47.0	–
∅Thy 8	26/M	Tetralogy of Fallot	20	complete	0.05	9.28	74.2	+

[a] CMV specific IgM was negative in all patients.
